# Delayed-onset Angioedema Following a Snakebite in a Patient on ACE Inhibitors: A Case Report

**DOI:** 10.5811/cpcem.1463

**Published:** 2023-06-26

**Authors:** Christopher Scott Sampson, Evan Schwarz

**Affiliations:** * University of Missouri School of Medicine, Department of Emergency Medicine, Columbia, Missouri; † Washington University School of Medicine in Saint Louis, Department of Emergency Medicine, Saint Louis, Missouri

**Keywords:** case report, angioedema, Crotalidae polyvalent immune fab, snakebite

## Abstract

**Introduction:**

Angiotensin converting enzyme inhibitors (ACEI) are a common class of medications prescribed to patients for hypertension. Anti-hypertensive use is not normally considered an important factor when treating patients with crotalid envenomations; however, in combination with the venom in this patient, it may have resulted in angioedema.

**Case Report:**

A 65-year-old male on ACEI presented to his community emergency department following a snake envenomation to his thumb. Six vials of Crotalidae polyvalent immune fab were administered, and he was transferred to a referral center. Approximately 18 hours after the envenomation, the patient complained of tongue swelling and difficulty speaking. There was evidence of angioedema, with the right side of the tongue significantly enlarged compared to the left. He was intubated for airway protection and remained on a ventilator for three days.

**Conclusion:**

Angiotensin converting enzyme inhibitors may potentiate the effects of exogenous bradykinin as some snake venom has naturally occurring bradykinin, which may further amplify its effects. Extra vigilance may be warranted for the development of angioedema in patients receiving ACEI.

## INTRODUCTION

Angiotensin converting enzyme inhibitors (ACEI) are a common class of medications prescribed to patients for hypertension. Their use in patients envenomated by North American crotalids is generally not considered to be clinically significant. We report a case of delayed angioedema potentially from an interaction of the venom and ACEI in a patient envenomated by a copperhead (*Agkistrodon contortix*).

## CASE REPORT

A 65-year-old male presented to a community emergency department (ED) following a snake envenomation to his thumb. Approximately four hours prior to arrival, he had attempted to pick up a copperhead in the field by his home and “it turned around and bit me on the thumb.” He complained of pain and swelling to thumb, hand, and wrist, along with shortness of breath. His past medical history included chronic obstructive pulmonary disease, atrial fibrillation, hypertension, mitral and tricuspid regurgitation, coronary artery disease, and diabetes. His daily oral medications included amiodarone 400 milligrams (mg), apixaban 5 mg, atorvastatin 40 mg, clopidogrel 75 mg, furosemide 20 mg, lisinopril 5 mg, and metoprolol succinate extended release 25 mg. He had been taking the lisinopril as prescribed for almost two years.

Initial vital signs were temperature of 36.5° Celsius, heart rate 74 beats per minute, blood pressure 128/82 millimeters of mercury (mm Hg), and respiratory rate 26 breaths per minute with saturations of 96% on room air. Initial examination was notable for wheezing bilaterally, an irregularly irregular heartbeat, and edema of the thumb, hand, and wrist without ecchymosis or necrosis. His white blood cell count was 10 × 10^9^ per liter (L) (reference range 3.50–10.50 × 10^9^/L), hemoglobin 13.7 grams per deciliter (g/dL) (reference range 12–15.5 g/dL), hematocrit 41.5% (reference range:34.9–44.5%), and platelets 163 × 10^9^/L (reference range 150–450 × 10^9^/L). Prothrombin time was 12.8 seconds (s) (reference range 12.5–15.1 s) with an international normalized ratio 1.09 (reference range 0.9–1.2), and partial thromboplastin time 27.9 s (reference range 25.8–35.6 s). Electrolytes were within normal limits.

An electrocardiogram demonstrated atrial fibrillation at a normal rate with no ischemic changes. His wheezing resolved with a nebulized albuterol breathing treatment. Shortly into his ED stay, the patient complained of increasing pain and swelling along with nausea. On re-examination, edema was noted up to the level of his elbow. Six vials of crotalidae polyvalent immune fab (FabAV) were administered intravenously (IV), and he was transferred to a referral center.

Upon arrival at the referral ED, the patient only had mild edema of his hand and wrist. Ink markings placed at the community ED and transport team were noted on his forearm and upper arm, with the edema not extending past the highest levels of the markings. Vital signs were normal. He was admitted overnight for serial examinations and analgesia with ketorolac and oxycodone.

Approximately 18 hours after the envenomation, the patient complained of tongue swelling and difficulty speaking. He did not develop new wheezing or a rash. There was evidence of angioedema with the right side of the tongue significantly enlarged when compared to the left side. The patient had difficulty controlling his secretions but could swallow his saliva without problem. However, his voice was muffled compared to when he arrived. He received famotidine 20 mg IV, methylprednisolone 125 mg IV, and 50 mg of IV diphenhydramine without improvement; 0.2 mg epinephrine was administered intramuscularly.

Otolaryngology performed a bedside rhinoscopy. Nasal mucosa was examined and was within normal limits. The nasopharynx, soft palate, and oropharynx were unremarkable. Fullness of the base of his tongue was noted. His epiglottis was normal with questionably minimal thickening; the remainder of the glottis and supraglottis were normal. True vocal cords were mobile bilaterally.

Given increasing symptoms, the patient was intubated without difficulty. He remained intubated for three days and was extubated without difficulty. The day after extubation, the patient complained of seeing bugs on the ventilator of his room overnight, as well as spiders and birds outside his window. He received benzodiazepines for either delirium vs alcohol withdrawal with improvement over the next 24 hours. Symptoms improved in less than 24 hours. He was discharged the next day.

## DISCUSSION

Copperheads are responsible for most native venomous snake envenomations in Missouri. While there are two species of rattlesnakes in this state, there are far fewer envenomations from rattlesnakes than copperheads here. Furthermore, given that the patient only experienced pain and swelling without necrosis or hematologic abnormalities, the envenomation is most consistent with that of a copperhead, particularly in this area.

**CPC-EM Capsule**

What do we already know about this clinical entity?

*Patients prescribed angiotensin converting enzyme inhibitors (ACEI) have a small risk of angioedema; ACEI mimic the action of viper venom, which prevents breakdown of bradykinin.*

What makes this presentation of disease reportable?

*A patient taking ACEI presented following a copperhead bite, received antivenom, and developed a delayed, severe case of angioedema.*

What is the major learning point?

*Angiotensin converting enzyme inhibitors may potentiate the effects of exogenous bradykinin, amplifying its effects.*

How might this improve emergency medicine practice?

*Extra vigilance may be warranted for the development of angioedema in patients with snakebites who are receiving ACEI.*

The signs and symptoms of crotalinae (pit viper) envenomation can vary from local to systemic. Localized complaints include pain, edema, erythema, and ecchymosis. These symptoms generally appear within eight hours of the envenomation; dry bites occur in approximately 25% of cases. Nausea, vomiting, diaphoresis and, very rarely, coagulopathy have been reported. Systemic complaints include nausea, vomiting, diaphoresis, coagulopathy, neurologic abnormalities, nephrotoxicity, angioedema, and increased vascular permeability.^1^–^2^ We could not find any reports in the literature of angioedema associated with a copperhead bite.

Crotalidae polyvalent immune fab is a sheep-derived antivenom created for management of crotalid envenomation. The medication is administered intravenously. Indications for administration include significant or progressive local tissue damage and evidence of hematological toxicity.^3^ A recent randomized controlled trial demonstrated the benefit of treating patients envenomated by copperheads with antivenom.^4^ Hypersensitivity including anaphylaxis is a known, although rare, complication following the administration of FabAV. The most common complications are urticaria, rash, nausea, pruritis, and back pain.^5^ Immediate serious adverse events related to FabAV include two cases of tongue swelling.

A review of the North American Snakebite Registry demonstrated that only 1.1% of patients developed a combination of hypotension, bronchospasm, and angioedema following administration of FabAV.^6^ Addition-ally, a 68-year-old male developed urticaria and hypotension during the initial 20 minutes of an infusion of six vials of FabAV, which temporarily resolved after he received diphenhydramine, famotidine, and methylpred-nisolone. The infusion was restarted and completed. However, shortly afterward, hypotension and urticaria recurred, and angioedema developed requiring epinephrine.^7^ A 36-year-old also developed angioedema while receiving FabAV. All reports of angioedema secondary to FabAV use have been immediate and occurred during the infusion period.^8^

Captopril, the original ACEI, was developed after peptides in the Brazilian viper *Bothrops jararaca*’s venom were discovered to inhibit angiotensin converting enzyme.^9^ Angiotensin converting enzyme inhibitors prevent the breakdown of bradykinin, which may lead to the development of angioedema. Components in the venom of a pit viper include histamine and bradykinin-like factors that can cause systemic effects.^10^ Given that the mechanism of angioedema from ACEI is thought to be through bradykinin release and that our patient had a pit viper envenomation, the delayed angioedema may have been a result of both synergistically interacting. While FabAV may cause angioedema, this is rare, and all reports of angioedema secondary to FabAV use has been immediate and occurred during the infusion period. Given the lack of unpublished reports and the time frame of onset, the angioedema was not likely associated with the antivenin infusion. Delayed-onset venom effects may also result in angioedema. However, this seems unlikely as it has not been previously reported.

A case report from Sweden described severe and prolonged hypertension in a 60-year-old male caused by an adder bite. The case report suggests that given ACEI potentiating the effects of exogenous bradykinin and that some snake’s venom has naturally occurring bradykinin which may amplify its effects.^11^ ACEI angioedema appears to occur through decreased bradykinin metabolism by the ACE enzyme.^12^ Components in the venom of a pit viper include histamine and bradykinin-like factors which can cause systemic effects.^10^ Given the mechanism of angioedema from ace inhibitors, the bradykinin accumulation from his medication and associated pit viper envenomation, the delayed angioedema may be a result of both synergistically interacting.

Thus, we believe that this patient most likely developed angioedema due to an amplification of bradykinin from his prescribed ACEI combined with bradykinin in the snake venom. Despite copperhead bites not being associated with angioedema, the levels of bradykinin were possibly increased, and angioedema developed. The patient had no reported previous episodes of angioedema and had been taking his ACEI for many years. While ACEI- associated angioedema can occur at any time, most cases occur within weeks of its initiation.

## CONCLUSION

In the setting of a crotalid envenomation in a patient on an ACE inhibitor, a delayed reaction may occur and should be included in patient education. In a patient on ACEI, extra vigilance may be warranted for the development of angioedema.
